# The Emerging Role of miRNAs and Their Clinical Implication in Biliary Tract Cancer

**DOI:** 10.1155/2016/9797410

**Published:** 2016-12-27

**Authors:** Nina Nayara Ferreira Martins, Kelly Cristina da Silva Oliveira, Amanda Braga Bona, Marília de Arruda Cardoso Smith, Geraldo Ishak, Paulo Pimentel Assumpção, Rommel Rodríguez Burbano, Danielle Queiroz Calcagno

**Affiliations:** ^1^Núcleo de Pesquisas em Oncologia, Universidade Federal do Pará, Belém, PA, Brazil; ^2^Laboratório de Citogenética Humana, Instituto de Ciências Biológicas, Universidade Federal do Pará, Belém, PA, Brazil; ^3^Universidade Federal de São Paulo, São Paulo, SP, Brazil

## Abstract

Biliary tract cancers are aggressive malignancies that include gallbladder cancer and tumors of intra- and extrahepatic ducts and have a poor prognosis. Surgical resection remains the main curative therapy. Nevertheless, numerous patients experience recurrence even after radical surgery. This scenario drives the research to identify biliary tract cancer biomarkers despite the limited progress that has been made. Recently, a large number of studies have demonstrated that deregulated expression of microRNAs is closely associated with cancer development and progression. In this review, we highlight the role and importance of microRNAs in biliary tract cancers with an emphasis on utilizing circulating microRNAs as potential biomarkers. Additionally, we report several single-nucleotide polymorphisms in* microRNA* genes that are associated with the susceptibility of biliary tract tumors.

## 1. Background

Despite their relatively rare incidence, biliary tract cancers (BTCs) are an aggressive tumor group with poor prognosis and are characterized by early lymph node and systemic metastases [1]. These tumors include gallbladder cancer (GBC) and cholangiocarcinoma (CCA), which is divided into intrahepatic cholangiocarcinoma (iCCA) and extrahepatic cholangiocarcinoma (eCCA). Currently, surgical resection remains the only curative treatment for BTCs, and neoadjuvant chemoradiotherapy is not a standard option for patients with these malignancies. Moreover, many cases present with recurrence even after radical surgery, and patients with recurrent or metastatic BTCs usually have a poor outcome [2]. Therefore, there is a need for additional investigations to determine potential biomarkers of BTCs for early diagnosis, determining patient prognosis and the development of targeted therapy.

Recent studies have described microRNAs (miRNAs) as potential biomarkers in different cancer types [3–6]. However, miRNA expression and their implications in the diagnosis of, prognosis of, and therapeutic applications towards BTCs remain elusive.

miRNAs are small noncoding RNAs (18–25 nucleotides) that play important roles in the regulation of a large number of essential biological functions that are critical to the development of different cancer types, including cell proliferation, differentiation, apoptosis, migration, and invasion [7].

miRNA biogenesis initiates in the nucleus, where miRNA genes are usually transcribed by RNA polymerase II, resulting in a primary transcript of miRNA (pri-miRNAs) [8]. During the initial processing of pri-miRNAs, the Drosha-DGCR8 complex cleaves the pri-miRNA, releasing a hairpin structure named pre-miRNA (~70 nucleotides). Pre-miRNAs are transferred to the cytoplasm and converted into an miRNA duplex by Exportin-5 and the Dicer-TRBP complex, respectively. Then, a helicase separates the double-stranded miRNA to produce one stable single-stranded miRNA, while the other strand is processed for autolytic degradation. The stable mature miRNA strand is loaded into the RNA-induced silencing complex (RISC) to mechanistically target the 3′ untranslated regions (3′UTRs) of protein coding mRNAs, thereby acting as posttranscriptional regulators by two mechanisms: mRNA degradation (when the sequences are perfect complements) and inhibition of translational initiation (when there is partial complementarily) [9]. Thus, miRNAs act as negative regulators of posttranscriptional gene expression of target mRNAs.

It has been well established that miRNAs could regulate approximately 60% of human genes, including many oncogenes and tumor suppressor genes; this phenomenon strengthens the importance of these noncoding RNAs as relevant regulators in cancer [10].

In this review, we focus on the roles and importance of miRNAs in BTCs and highlight the potential of circulating miRNAs as diagnostic and prognostic biomarkers. Therefore, we reported several single-nucleotide polymorphisms (SNPs) in* miRNA* genes associated with BTC susceptibility.

## 2. Roles and Clinical Significance of miRNAs in BTCs

A large number of deregulated miRNAs have been categorized as oncomiRs (oncogene miRNAs) and/or tsmiRs (tumor suppressor miRNAs) in cancer depending on the effect of the target mRNA.

In BTCs, several studies on miRNA expression have identified many upregulated oncomiRs and downregulated tsmiRs as well as their potential targets (Table 1).

One of the best-described miRNAs in BTCs is hsa-miR-21, which is usually identified as an oncomiR since its overexpression has been associated with invasion and metastasis [11–19, 21–25, 27, 30, 41, 47–50]. Liu et al. [13] observed that overexpression of hsa-miR-21 significantly promotes cell migration, invasion, and xenograft growth after transfection of hsa-miR-21 into CCA cell lines (QBC939 and RBE). Moreover, these authors showed decreased E-cadherin expression and increased N-cadherin and vimentin expression after hsa-miR-21 overexpression. Thus, hsa-miR-21 could induce the epithelial-mesenchymal transition (EMT) in CCA. In this process, epithelial cells lose their cell polarity and cell adhesion—probably due to the decrease of E-cadherin expression—which allows cells to migrate and invade surrounding tissues; this the loss of E-cadherin expression plays a key role in tumor invasion and metastasis.

Similarly, aberrant expression of miRNAs also induces EMT and enhances the metastatic potential of GBC cells [24, 51]. Bao et al. [51] reported that hsa-miR-101 overexpression inhibits the proliferation, migration, and invasion of GBC cells, induces the increased expression of E-cadherin and *β*-catenin, and causes decreased expression of vimentin. Furthermore, these authors observed that hsa-miR-101 downregulation was correlated with tumor size, invasion, lymph node metastasis, TNM stage, and poor survival in GBC patients. These results indicate that hsa-miR-101 plays a tsmiR role and attenuates EMT and metastasis in GBC.

Accumulating evidence has indicated that hsa-miR-146b-5p presents critical tumor suppressor properties [52, 53]. Its expression was significantly downregulated in GBC tissue compared with adjacent nonneoplastic tissues. In addition, the overexpression of hsa-miR-146b-5p in the SGC-996 GBC cell line inhibited cell growth by enhancing apoptosis and arresting the cells at G1 phase. However, the enforced expression of* EGFR*, a cell surface protein that binds to epidermal growth factor (which inducing cell proliferation), reversed the ability of hsa-miR-146b-5p to inhibit proliferation. Moreover, hsa-miR-146b-5p expression levels were significantly correlated with tumor size and cancer progression [46].

Recent studies have described hsa-miR-135a-5p as having a tsmiR role [54–56]. In GBC, Zhou et al. [44] found that hsa-miR-135a-5p levels were significantly downregulated in tumors compared to adjacent nontumor gallbladder tissues and were correlated with neoplasms of histological grades III and IV. Additionally, this study identified* VLDLR* as a direct and functional target gene of hsa-miR-135a-5p in GBC tissues. Furthermore, the transfection with a hsa-miR-135a-5p mimetic inhibited the proliferative and colony-forming abilities of GBC cells by arresting the cells in G1/S phase. These data suggest that hsa-miR-135a-5p may inhibit the proliferation of GBC cells.

## 3. Circulating miRNAs as Potential BTCs Biomarkers

Several studies have reported that detectable miRNAs in bodily fluids (e.g., plasma, serum, urine, and saliva) are more stable in comparison with other circulating nucleic acids [57]. Therefore, circulating miRNAs may be noninvasive and specific diagnostic and/or prognostic molecular biomarkers for human diseases, including cancer [4, 7, 58, 59]. In BTCs, many circulating miRNAs seem to be reproducible and reliable potential biomarkers as well as possible therapeutic targets [60].  Table 2 summarizes the circulating miRNAs with potential diagnostic, prognostic, and predictive biomarker applications in BTCs.

In CCA patients, Cheng et al. [64] observed different expression levels of circulating hsa-miR-106a not only between CCAs and healthy controls but also among CCAs and benign bile duct diseases (e.g., primary bile duct stone and congenital biliary duct cysts). Furthermore, they identified decreased hsa-miR-106a levels in patients with lymph node metastasis compared with those without metastasis, indicating the possible role of hsa-miR-106a in the occurrence of lymph node metastasis.

Interestingly, Voigtländer et al. [65] found a distinct circulating miRNA profile in the bile and serum samples from CCA patients and patients with primary sclerosing cholangitis (PSC), a noncancerous disease. Furthermore, bile samples from patients with concomitant PSC and CCA (PSC/CCA) were also included in this study. Their results showed higher expression levels of hsa-miR-126, hsa-miR-26a, hsa-miR-30b, hsa-miR-122, and hsa-miR-1281 in PSC patients than those in CCA patients. However, bile samples showed hsa-miR-640, hsa-miR-1537, and hsa-miR-3189 downregulation, as well as hsa-miR-412 upregulation in PSC and PSC/CCA patients. These results demonstrated that PSC and CCA patients have distinct miRNA profiles in their bile and serum, which could be used to discriminate these diseases.

A small number of studies have described circulating miRNAs in patients with GBC. Kishimoto et al. [62] demonstrated an increase in the hsa-miR-21 expression levels in plasma from GBC patients before curative resection when compared with postsurgical patients and healthy volunteers. These findings suggest that hsa-miR-21 plasma levels were significantly affected by cancer occurrence and might have the potential to be a diagnostic biomarker for GBC patients.

Recently, Li and Pu [47] described significantly deregulated miRNAs in the peripheral blood samples of GBC patients compared with healthy volunteers. The expression levels of hsa-miR-187, hsa-miR-192, and hsa-miR-202 were upregulated while hsa-miR-143 was downregulated. These results were associated with lymph node metastasis, inflammation, immune reaction, and poor prognosis and could be translated to clinical practice as biomarkers for the early diagnosis, prognosis, and predictive response in patients with GBC.

Although most studies involving circulating miRNAs utilize real-time PCR for detection, Kojima et al. [68] used a highly sensitive microarray denoted as “3D Gene” that was capable of simultaneously analyzing more than 2,500 miRNAs in serum samples from patients with pancreato-biliary cancers. These authors found several significantly dysregulated miRNAs, including 30 upregulated miRNAs and 36 downregulated miRNAs in BTCs. However, none of these miRNAs could be used as single biomarker for this type of cancer. The best results were achieved with a panel of eight miRNAs (hsa-miR-6075, hsa-miR-4294, hsa-miR-6880-5p, hsa-miR-6799-5p, hsa-miR-125a-3p, hsa-miR-4530, hsa-miR-6836-3p, and hsa-miR-4476).

## 4. *miRNA* Single-Nucleotide Polymorphisms in BTCs

In general, aberrations in miRNA expression result from either epigenetic modifications or genomic changes, which include chromosomal rearrangements, mutations, or SNPs [69].

Several SNPs in miRNAs can lead to distinctions in the miRNA expression levels, which can modulate miRNA-target gene expression and, subsequently, affect cancer susceptibility [4, 70]. However, few studies have been performed to identify SNPs in miRNAs in BTCs patients until now.

The SNPs hsa-miR-27a rs895819, hsa-miR-570 rs4143815, and hsa-miR-181a rs12537 have been found to play important roles in many cancer types [71–78], and their contribution in BTCs has been explored. Gupta et al. [70] observed that the combination of hsa-miR-27a rs895819, hsa-miR-570 rs4143815, and hsa-miR-181a rs12537 was the best gene-gene interaction model for predicting the susceptibility and treatment response in GBC patients. Moreover, the SNPs hsa-miR-27a rs895819 and hsa-miR-181a rs12537 were associated with treatment toxicity but had no influence on the survival outcomes of GBC patients with locally advanced and/or metastatic tumors.

SNPs in pri-miRNAs and pre-miRNAs could also affect miRNA processing, miRNA expression, and cancer susceptibility. Srivastava et al. [79] reported genetic polymorphisms in pre-mir-196a2 rs11614913 (C>T), pre-hsa-mir-196a rs11614913, and pre-hsa-mir-499 rs3746444 (T>C) that were associated with an increased overall risk of developing GBC development. In CCA, Mihalache et al. [80] investigated* *the G/C variant in* *pre-hsa-miR-146a rs2910164 and found no significant relationship between genetic susceptibility and CCA.

Additional studies addressing the identification of miRNA SNPs could be useful to assess the individual susceptibility of BTCs and improve our understanding of their potential contribution to the disease as well as aid in the development of potential clinical applications.

## 5. Conclusion

miRNAs are profoundly involved in tumor onset and progression [81–84]. However, the implications of miRNA for the diagnosis, prognosis, and therapeutic options for patients with BTCs remain unsatisfactory. This review highlighted some miRNAs that are dysregulated in BTCs, their targets, and the possible clinical implications. A better understanding of the therapeutic applications of miRNAs could lead to future clinical trials involving the inhibition of oncomiRs or the promotion of expression of tsmiRs as new approaches against diverse cancer types, including aggressive BTCs.

Here, we also reported several circulating miRNAs as possible diagnostic, prognostic, and/or predictive biomarkers in BTCs. Circulating miRNAs could be promising potential biomarkers for cancers because detectable miRNAs in the bodily fluids are stable and can be measured using noninvasive methods [57]. BTCs are usually asymptomatic; therefore, the use of miRNAs as early diagnostic biomarkers could be a useful tool to improve the long-term survival of BTC patients. However, more studies with clinical outcomes are needed to identify which miRNAs could serve as either a potential therapeutic target or diagnostic and prognostic biomarkers of BTCs.

Moreover, several SNPs in miRNAs can affect the expression of target genes, leading to a cellular disorder and, consequently, tumorigenesis [4, 70]. However, few studies have been performed to identify SNPs in the miRNAs expressed by BTC patients until now; this review emphasizes the need to expand the knowledge in this field of study.

## Figures and Tables

**Table 1:** Deregulated miRNAs in BTCs.

miRNA	Tumor	Expression	Target	Roles in BTCs	Reference
hsa-miR-21	CCA	↑	*PTEN*	Invasion Migration Chemoresistance	[11–14]
		↓	*TPM1*	DNA methylation Histone deacetylation	[15]
		↓	*15-PGDH*	Inflammation	[16]
			*HPGD*		
		↑	*PDCD4*	Lymph node metastasis Migration	[17–20]
		↑	*RECK*	Migration Metastasis	[21, 22]
		↑	*TIMP3*	Apoptosis	[18]
		↑	*—*	pTNM Prognosis	[23]

hsa-miR-20^a^	GBC	↑	*SMAD7*	Invasion Metastasis Migration Prognosis	[24]

hsa-miR-34a	CCA	↓	*C-MYC*	Progression	[25]
	GBC	↓	*PNUTS*	Proliferation Prognosis	[26]

hsa-miR-335	GBC	↓	*BMI1*	Invasion Lymph node metastasis pTNM Prognosis	[27, 28]

hsa-miR-148a	CCA	↓	*DNMT1*	Prognosis	[29]

hsa-miR-31	CCA	↑	*RASA1*	Apoptosis Proliferation	[30]

hsa-miR-200b/c	CCA	↓	*SUZ12*	Chemoresistance Invasion Migration	[28]

hsa-miR-210	CCA	↑	*MNT*	Progression	[25]

Let-7a	CCA	↓	*RAS*	Progression	[21]

hsa-miR-370	CCA	↓	*MAP3K8*	Inflammation pTNM	[31]

hsa-miR-29b	CCA	↓	*C-MYC*	Apoptosis	[32]

hsa-miR-101	CCA	↓	*VEGF*	Angiogenesis	[33]
			*COX-2*		

hsa-miR-200b/c	CCA	↓	*ROCK2*	Migration	[28]

hsa-miR-138	CCA	↓	*RHOC*	Migration	[34]

hsa-miR-376c	CCA	↓	*GRB2*	Migration	[35]

hsa-miR-124	CCA	↓	*SMYD3*	Migration	[36]

hsa-miR-204	CCA	↓	*SLUG*	Migration	[37]

hsa-miR-214	CCA	↓	*TWIST*	Migration	[38]

hsa-miR-200c	CCA	↓	*NCAM1*	Migration	[39]

hsa-miR-200b	CCA	↑	*PTPN12*	Chemoresistance	[14]

hsa-miR-29b	CCA	↓	*PIK3R1*	Chemoresistance	[40]
			*MMP2*		
hsa-miR-205	CCA	↓	*—*	Chemoresistance	
hsa-miR-221	CCA	↓	*PIK3R1*	Chemoresistance	

hsa-miR-182	GBC	↑	*CADM1*	Invasion Migration Metastasis	[27]

hsa-miR-155	GBC	↑	*SMAD7*	Invasion Lymph node metastasis Proliferation Prognosis	[41]

hsa-miR-130a	GBC	↓	*HOTAIR*	Invasion Proliferation	[42]

hsa-miR-26a	GBC	↓	*HMGA2*	pTNM Proliferation	[43]

hsa-miR-135a-5p	GBC	↓	*VLDLR*	pTNM Proliferation	[44]

hsa-miR-218-5p	GBC	↓	*BMI1*	Invasion Migration Proliferation	[45]

hsa-miR-146-5p	GBC	↓	*EGFR*	Apoptosis pTNM Proliferation	[46]

hsa-miR-1	GBC	↓	*VEGF-A*	Apoptosis Proliferation	[47]
			*AXL*		
hsa-miR-145	GBC		*AXL*		
hsa-miR-143	GBC	↓	*AXL*	Lymph node metastasis pTNM stage	
hsa-miR-122 hsa-miR-187	GBC	↑			

**Table 2:** Circulating miRNAs in patients with BTC as potential diagnostic, prognostic, and predictive biomarkers.

miRNA	Expression	Samples	N samples	Potential biomarker	Method	Clinical implication	Reference
hsa-miR-9	↑	Bile	BTCs (9) HV (9)	Diagnostic Prognostic	RT-PCR	Metastasis	[61]

hsa-miR-145	↑	Bile	BTCs (9) HV (9)	Diagnostic	RT-PCR	—	[61]

hsa-miR-21	↑	Plasma	BTCs (94) HV (50) BBD (2)	Diagnostic	qRT-PCR	Inflammatory reaction	[62]
		Peripheral blood	GBC (40) HV (40)	Diagnostic	qRT-PCR	—	[47]

hsa-miR-150	↑	Plasma	iCCA (15)	Diagnostic	qRT-PCR	Tumor progression	[63]

hsa-miR-106a	↓	Serum	CCA (103)	Prognostic	qRT-PCR	Lymph node metastasis	[64]
			HV (20)				

hsa-miR-126	↑	Serum	PSC (40) CCA (31) HV (12)	Diagnostic	RT-PCR	—	[65]

hsa-miR-26a	↑	Serum	PSC (40) CCA (31) HV (12)	Diagnostic	RT-PCR	—	[65]

hsa-miR-30b	↑	Serum	PSC (40) CC (31) HV (12)	Diagnostic	RT-PCR	—	[65]

hsa- miR-122	↑	Serum	PSC (40) CC (31) HV (12)	Diagnostic	RT-PCR	—	[65]

hsa-miR-1281	↑	Serum	PSC (40) CC (31) HV (12)	Diagnostic	RT-PCR	—	[65]

hsa-miR -187	↑	Peripheral blood	GBC (40) HV (40)	Diagnostic Prognostic Predictive	qRT-PCR	Lymph node metastasis Poor prognosis	[47]

hsa-miR-192	↑	Peripheral blood	GBC (40) HV (40)	Diagnostic Prognostic Predictive	qRT-PCR	Inflammatory reaction Immune reaction Lymph node metastasis	[47]
		Serum	iCCA (11) HV (09)	Diagnostic Prognostic	miRNA RT-PCR array	Lymph node metastasis Poor prognosis	[66]

hsa-miR-194	↑	Serum	CCA (70) HV (70)	Diagnostic	qRT-PCR	Tumor progression	[58]

hsa-miR -202	↑	Peripheral blood	GBC (40) HV (40)	Diagnostic Prognostic Predictive	qRT-PCR	Lymph node metastasis	[47]

hsa-let- 7a	↓	Peripheral blood	GBC (40) HV (40)	Diagnostic	qRT-PCR	—	[47]

hsa-miR -143	↓	Peripheral blood	GBC (40) HV (40)	Diagnostic Prognostic Predictive	qRT-PCR	Inflammatory and immune reaction Lymph node metastasis	[47]

hsa-miR-335	↓	Peripheral blood	GBC (40) HV (40)	Diagnostic	qRT-PCR	—	[47]

hsa-miR-1307-3p	↓	Plasma	iCCA (13) HV (5)	Diagnostic	qRT-PCR	—	[67]

hsa-miR-1275	↑	Plasma	iCCA (13) HV (5)	Diagnostic	qRT-PCR	—	[67]

hsa- miR-320b	↑	Plasma	iCCA (13) HVs (5)	Diagnostic	qRT-PCR	—	[67]

hsa-miR-874	↑	Plasma	iCCA (13) HVs (5)	Diagnostic	qRT-PCR	—	[67]

hsa-miR-483-5p	↑	Plasma	iCCA (13) HV (5)	Diagnostic	qRT-PCR	—	[67]
		Serum	CCA (70) HV (70)	Diagnostic	qRT-PCR	Tumor progression	[58]

hsa-miR-885-5p	↑	Plasma	iCCA (13) HV (5)	Diagnostic	qRT-PCR	—	[67]

hsa-miR-92b-3p	↑	Plasma	iCCA (13) HV (5)	Diagnostic	qRT-PCR	—	[67]

hsa-miR-505-3p	↑	Plasma	iCCA (13) HV (5)	Diagnostic	qRT-PCR	—	[67]

hsa-miR-6836-3p	↑	Serum	BTCs (98) HV (150)	Diagnostic	3D-Gene	—	[68]

hsa-miR-6075	↑	Serum	BTCs (98) HV (150)	Diagnostic	3D-Gene	—	[68]

hsa- miR-4634	↑	Serum	BTCs (98) HV (150)	Diagnostic	3D-Gene	—	[68]

hsa-miR-4294	↓	Serum	BTCs (98) HV (150)	Diagnostic	3D-Gene	—	[68]

hsa-miR-6880-5p	↓	Serum	BTCs (98) HV (150)	Diagnostic	3D-Gene	—	[68]

hsa-miR-6799-5p	↓	Serum	BTCs (98) HV (150)	Diagnostic	3D-Gene	—	[68]

hsa-miR-125a-3p,	↓	Serum	BTCs (98) HV (150)	Diagnostic	3D-Gene	Tumor progression	[68]

hsa-miR-4530	↓	Serum	BTCs (98) HV (150)	Diagnostic	3D-Gene	—	[68]

hsa-miR-7114-5p	↓	Serum	BTCs (98) HV (150)	Diagnostic	3D-Gene	—	[68]

hsa-miR-4476	↓	Serum	BTCs (98) HV (150)	Diagnostic	3D-Gene	—	[68]

BBD: benign biliary disorders; BTCs: biliary tract cancers; CCA: cholangiocarcinoma; iCCA: intrahepatic cholangiocarcinoma; GBC: gallbladder cancer; HV: heath volunteers; PSC: primary sclerosing cholangitis.

## References

[1] Yoo K. H., Kim N. K. D., Kwon W. I. (2016). Genomic alterations in biliary tract cancer using targeted sequencing. *Translational Oncology*.

[2] Ciombor K. K., Goff L. W. (2013). Advances in the management of biliary tract cancers. *Clinical Advances in Hematology and Oncology*.

[3] Gigek C. O., Chen E. S., Calcagno D. Q., Wisnieski F., Burbano R. R., Smith M. A. C. (2012). Epigenetic mechanisms in gastric cancer. *Epigenomics*.

[4] Calcagno D. Q., Cardoso Smith M. D. A., Burbano R. R. (2015). Cancer type-specific epigenetic changes: gastric cancer. *Methods in Molecular Biology*.

[5] Saumet A., Lecellier C.-H. (2015). MicroRNAs and personalized medicine: evaluating their potential as cancer biomarkers. *Advances in Experimental Medicine and Biology*.

[6] Huo D., Clayton W. M., Yoshimatsu T. F., Chen J., Olopade O. I. (2016). Identification of a circulating MicroRNA signature to distinguish recurrence in breast cancer patients. *Oncotarget*.

[7] Yang G., Zhang L., Li R., Wang L. (2016). The role of microRNAs in gallbladder cancer (Review). *Molecular and Clinical Oncology*.

[8] Lee Y., Kim M., Han J. (2004). MicroRNA genes are transcribed by RNA polymerase II. *EMBO Journal*.

[9] Lin S., Gregory R. I. (2015). MicroRNA biogenesis pathways in cancer. *Nature Reviews Cancer*.

[10] Weber C. (2013). MicroRNAs: from basic mechanisms to clinical application in cardiovascular medicine. *Arteriosclerosis, Thrombosis, and Vascular Biology*.

[11] Zhang J., Jiao J., Cermelli S. (2015). miR-21 inhibition reduces liver fibrosis and prevents tumor development by inducing apoptosis of CD24^+^ progenitor cells. *Cancer Research*.

[12] He Q., Cai L., Shuai L. (2013). Ars2 is overexpressed in human cholangiocarcinomas and its depletion increases PTEN and PDCD4 by decreasing microRNA-21. *Molecular Carcinogenesis*.

[13] Liu Z., Jin Z.-Y., Liu C.-H., Xie F., Lin X.-S., Huang Q. (2015). MicroRNA-21 regulates biological behavior by inducing EMT in human cholangiocarcinoma. *International Journal of Clinical and Experimental Pathology*.

[14] Meng F., Henson R., Lang M. (2006). Involvement of human micro-RNA in growth and response to chemotherapy in human cholangiocarcinoma cell lines. *Gastroenterology*.

[15] Yang W., Wang X., Zheng W., Li K., Liu H., Sun Y. (2013). Genetic and epigenetic alterations are involved in the regulation of TPM1 in cholangiocarcinoma. *International Journal of Oncology*.

[16] Lu L., Byrnes K., Han C., Wang Y., Wu T. (2014). MiR-21 targets 15-PGDH and promotes cholangiocarcinoma growth. *Molecular Cancer Research*.

[17] Chusorn P., Namwat N., Loilome W. (2013). Overexpression of microRNA-21 regulating PDCD4 during tumorigenesis of liver fluke-associated cholangiocarcinoma contributes to tumor growth and metastasis. *Tumor Biology*.

[18] Selaru F. M., Olaru A. V., Kan T. (2009). MicroRNA-21 is overexpressed in human cholangiocarcinoma and regulates programmed cell death 4 and tissue inhibitor of metalloproteinase 3. *Hepatology*.

[19] Liu C.-Z., Liu W., Zheng Y. (2012). PTEN and PDCD4 are bona fide targets of microRNA-21 in human cholangiocarcinoma. *Chinese Medical Sciences Journal*.

[20] He J., Yue Y., Dong C., Xiong S. (2013). MiR-21 confers resistance against CVB3-induced myocarditis by inhibiting PDCD4-mediated apoptosis. *Clinical and Investigative Medicine*.

[21] Namwat N., Chusorn P., Loilome W. (2012). Expression profiles of oncomir miR-21 and tumor suppressor let-7a in the progression of opisthorchiasis-associated cholangiocarcinoma. *Asian Pacific Journal of Cancer Prevention*.

[22] Huang Q., Liu L., Liu C.-H. (2013). MicroRNA-21 Regulates the invasion and metastasis in cholangiocarcinoma and may be a potential biomarker for cancer prognosis. *Asian Pacific Journal of Cancer Prevention*.

[23] Karakatsanis A., Papaconstantinou I., Gazouli M., Lyberopoulou A., Polymeneas G., Voros D. (2013). Expression of microRNAs, miR-21, miR-31, miR-122, miR-145, miR-146a, miR-200c, miR-221, miR-222, and miR-223 in patients with hepatocellular carcinoma or intrahepatic cholangiocarcinoma and its prognostic significance. *Molecular Carcinogenesis*.

[24] Chang Y., Yang J., Liu G. (2013). MiR-20a triggers metastasis of gallbladder carcinoma. *Journal of Hepatology*.

[25] Yang H., Li T. W. H., Peng J. (2011). A mouse model of cholestasis-associated cholangiocarcinoma and transcription factors involved in progression. *Gastroenterology*.

[26] Jin K., Xiang Y., Tang J. (2014). miR-34 is associated with poor prognosis of patients with gallbladder cancer through regulating telomere length in tumor stem cells. *Tumor Biology*.

[27] Qiu Y., Luo X., Kan T. (2014). TGF-*β* upregulates miR-182 expression to promote gallbladder cancer metastasis by targeting CADM1. *Molecular BioSystems*.

[28] Peng F., Jiang J., Yu Y. (2013). Direct targeting of SUZ12/ROCK2 by miR-200b/c inhibits cholangiocarcinoma tumourigenesis and metastasis. *British Journal of Cancer*.

[29] Braconi C., Huang N., Patel T. (2010). Microrna-dependent regulation of DNA methyltransferase-1 and tumor suppressor gene expression by interleukin-6 in human malignant cholangiocytes. *Hepatology*.

[30] Hu C., Huang F., Deng G., Nie W., Huang W., Zeng X. (2013). miR-31 promotes oncogenesis in intrahepatic cholangiocarcinoma cells via the direct suppression of RASA1. *Experimental and Therapeutic Medicine*.

[31] Meng F., Wehbe-Janek H., Henson R., Smith H., Patel T. (2008). Epigenetic regulation of microRNA-370 by interleukin-6 in malignant human cholangiocytes. *Oncogene*.

[32] Mott J. L., Kurita S., Cazanave S. C., Bronk S. F., Werneburg N. W., Fernandez-Zapico M. E. (2010). Transcriptional suppression of mir-29b-1/mir-29a promoter by c-Myc, hedgehog, and NF-kappaB. *Journal of Cellular Biochemistry*.

[33] Zhang J., Han C., Zhu H., Song K., Wu T. (2013). MiR-101 inhibits cholangiocarcinoma angiogenesis through targeting vascular endothelial growth factor (VEGF). *American Journal of Pathology*.

[34] Wang Q., Tang H., Yin S., Dong C. (2013). Downregulation of microRNA-138 enhances the proliferation, migrationand invasion of cholangiocarcinoma cells through the upregulation of RhoC/p-ERK/MMP-2/MMP-9. *Oncology Reports*.

[35] Iwaki J., Kikuchi K., Mizuguchi Y. (2013). MiR-376c down-regulation accelerates EGF-dependent migration by targeting GRB2 in the HuCCT1 human intrahepatic cholangiocarcinoma cell line. *PLoS ONE*.

[36] Zeng B., Li Z., Chen R. (2012). Epigenetic regulation of miR-124 by Hepatitis C Virus core protein promotes migration and invasion of intrahepatic cholangiocarcinoma cells by targeting SMYD3. *FEBS Letters*.

[37] Qiu Y.-H., Wei Y.-P., Shen N.-J. (2013). MiR-204 Inhibits epithelial to mesenchymal transition by targeting slug in intrahepatic cholangiocarcinoma cells. *Cellular Physiology and Biochemistry*.

[38] Li B., Han Q., Zhu Y., Yu Y., Wang J., Jiang X. (2012). Down-regulation of miR-214 contributes to intrahepatic cholangiocarcinoma metastasis by targeting Twist. *FEBS Journal*.

[39] Oishi N., Kumar M. R., Roessler S. (2012). Transcriptomic profiling reveals hepatic stem-like gene signatures and interplay of miR-200c and epithelial-mesenchymal transition in intrahepatic cholangiocarcinoma. *Hepatology*.

[40] Okamoto K., Miyoshi K., Murawaki Y. (2013). miR-29b, miR-205 and miR-221 enhance chemosensitivity to gemcitabine in HuH28 human cholangiocarcinoma cells. *PLoS ONE*.

[41] Kono H., Nakamura M., Ohtsuka T. (2013). High expression of microRNA-155 is associated with the aggressive malignant behavior of gallbladder carcinoma. *Oncology reports*.

[42] Ma M.-Z., Li C.-X., Zhang Y. (2014). Long non-coding RNA HOTAIR, a c-Myc activated driver of malignancy, negatively regulates miRNA-130a in gallbladder cancer. *Molecular Cancer*.

[43] Zhou H., Guo W., Zhao Y. (2014). MicroRNA-26a acts as a tumor suppressor inhibiting gallbladder cancer cell proliferation by directly targeting HMGA2. *International Journal of Oncology*.

[44] Zhou H., Guo W., Zhao Y. (2014). MicroRNA-135a acts as a putative tumor suppressor by directly targeting very low density lipoprotein receptor in human gallbladder cancer. *Cancer Science*.

[45] Ma M.-Z., Chu B.-F., Zhang Y. (2015). Long non-coding RNA CCAT1 promotes gallbladder cancer development via negative modulation of miRNA-218-5p. *Cell Death and Disease*.

[46] Cai J., Xu L., Cai Z., Wang J., Zhou B., Hu H. (2015). MicroRNA-146b-5p inhibits the growth of gallbladder carcinoma by targeting epidermal growth factor receptor. *Molecular Medicine Reports*.

[47] Li G., Pu Y. (2015). MicroRNA signatures in total peripheral blood of gallbladder cancer patients. *Tumor Biology*.

[48] Kitamura T., Connolly K., Ruffino L. (2012). The therapeutic effect of histone deacetylase inhibitor PCI-24781 on gallbladder carcinoma in BK5.erbB2 mice. *Journal of Hepatology*.

[49] Wang L.-J., He C.-C., Sui X. (2015). MiR-21 promotes intrahepatic cholangiocarcinoma proliferation and growth in vitro and in vivo by targeting PTPN14 and PTEN. *Oncotarget*.

[50] Sekine S., Shimada Y., Nagata T. (2013). Role of aquaporin-5 in gallbladder carcinoma. *European Surgical Research*.

[51] Bao R., Shu Y., Hu Y. (2016). miR-101 targeting ZFX suppresses tumor proliferation and metastasis by regulating the MAPK/Erk and smad pathways in gallbladder carcinoma. *Oncotarget*.

[52] Wu P. Y., Zhang X. D., Zhu J., Guo X. Y., Wang J. F. (2014). Low expression of microRNA-146b-5p and microRNA-320d predicts poor outcome of large B-cell lymphoma treated with cyclophosphamide, doxorubicin, vincristine, and prednisone. *Human Pathology*.

[53] Shen C., Yang H., Liu H., Wang X., Zhang Y., Xu R. (2015). Inhibitory effect and mechanisms of microRNA-146b-5p on the proliferation and metastatic potential of Caski human cervical cancer cells. *Molecular Medicine Reports*.

[54] Dang Z., Xu W.-H., Lu P. (2014). MicroRNA-135a inhibits cell proliferation by targeting Bmi1 in pancreatic ductal adenocarcinoma. *International Journal of Biological Sciences*.

[55] Shin J.-Y., Kim Y.-I., Cho S.-J. (2014). MicroRNA 135a suppresses lymph node metastasis through down-regulation of ROCK1 in early gastric cancer. *PLoS ONE*.

[56] Tang W., Jiang Y., Mu X., Xu L., Cheng W., Wang X. (2014). MiR-135a functions as a tumor suppressor in epithelial ovarian cancer and regulates HOXA10 expression. *Cellular Signalling*.

[57] Igaz I., Igaz P. (2015). Diagnostic relevance of microRNAs in other body fluids including urine, feces, and saliva. *EXS*.

[58] Bernuzzi F., Marabita F., Lleo A. (2016). Serum microRNAs as novel biomarkers for primary sclerosing cholangitis and cholangiocarcinoma. *Clinical & Experimental Immunology*.

[59] Piontek K., Selaru F. M. (2015). MicroRNAs in the biology and diagnosis of cholangiocarcinoma. *Seminars in Liver Disease*.

[60] Letelier P., Riquelme I., Hernández A., Guzmán N., Farías J., Roa J. (2016). Circulating MicroRNAs as biomarkers in biliary tract cancers. *International Journal of Molecular Sciences*.

[61] Shigehara K., Yokomuro S., Ishibashi O. (2011). Real-time PCR-based analysis of the human bile micrornaome identifies miR-9 as a potential diagnostic biomarker for biliary tract cancer. *PLoS ONE*.

[62] Kishimoto T., Eguchi H., Nagano H. (2013). Plasma miR-21 is a novel diagnostic biomarker for biliary tract cancer. *Cancer Science*.

[63] Wang S., Yin J., Li T. (2015). Upregulated circulating miR-150 is associated with the risk of intrahepatic cholangiocarcinoma. *Oncology Reports*.

[64] Cheng Q., Feng F., Zhu L. (2015). Circulating miR-106a is a novel prognostic and lymph node metastasis indicator for cholangiocarcinoma. *Scientific Reports*.

[65] Voigtländer T., Gupta S. K., Thum S. (2015). MicroRNAs in serum and bile of patients with primary sclerosing cholangitis and/or cholangiocarcinoma. *PLoS ONE*.

[66] Silakit R., Loilome W., Yongvanit P. (2014). Circulating miR-192 in liver fluke-associated cholangiocarcinoma patients: a prospective prognostic indicator. *Journal of Hepato-Biliary-Pancreatic Sciences*.

[67] Plieskatt J., Rinaldi G., Feng Y. (2015). A microRNA profile associated with Opisthorchis viverrini-induced cholangiocarcinoma in tissue and plasma. *BMC Cancer*.

[68] Kojima M., Sudo H., Kawauchi J. (2015). MicroRNA markers for the diagnosis of pancreatic and biliary-tract cancers. *PLoS ONE*.

[69] Chandra A., Ray A., Senapati S., Chatterjee R. (2015). Genetic and epigenetic basis of psoriasis pathogenesis. *Molecular Immunology*.

[70] Gupta A., Sharma A., Yadav A. (2015). Evaluation of miR-27a, miR-181a, and miR-570 genetic variants with gallbladder cancer susceptibility and treatment outcome in a north indian population. *Molecular Diagnosis and Therapy*.

[71] Sun Q., Gu H., Zeng Y. (2010). Hsa-mir-27a genetic variant contributes to gastric cancer susceptibility through affecting miR-27a and target gene expression. *Cancer Science*.

[72] Xu J., Yin Z., Shen H. (2013). A genetic polymorphism in pre-miR-27a confers clinical outcome of non-small cell lung cancer in a Chinese population. *PLoS ONE*.

[73] Wang Z., Lai J., Wang Y., Nie W., Guan X. (2013). The Hsa-miR-27a rs895819 (A>G) polymorphism and cancer susceptibility. *Gene*.

[74] Lin Y., Nie Y., Zhao J. (2012). Genetic polymorphism at miR-181a binding site contributes to gastric cancer susceptibility. *Carcinogenesis*.

[75] Ma J.-Y., Yan H.-J., Yang Z.-H., Gu W. (2015). Rs895819 within miR-27a might be involved in development of non small cell lung cancer in the Chinese Han population. *Asian Pacific Journal of Cancer Prevention*.

[76] Wang Z., Sun X., Wang Y., Liu X., Xuan Y., Hu S. (2014). Association between miR-27a genetic variants and susceptibility to colorectal cancer. *Diagnostic Pathology*.

[77] Deng Y., Bai H., Hu H. (2015). rs11671784 G/A variation in miR-27a decreases chemo-sensitivity of bladder cancer by decreasing miR-27a and increasing the target RUNX-1 expression. *Biochemical and Biophysical Research Communications*.

[78] Shi D., Li P., Ma L. (2012). A genetic variant in pre-miR-27a is associated with a reduced renal cell cancer risk in a Chinese population. *PLoS ONE*.

[79] Srivastava K., Srivastava A., Mittal B. (2010). Common genetic variants in pre-microRNAs and risk of gallbladder cancer in North Indian population. *Journal of Human Genetics*.

[80] Mihalache F., Höblinger A., Acalovschi M., Sauerbruch T., Lammert F., Zimmer V. (2012). A common variant in the precursor miR-146a sequence does not predispose to cholangiocarcinoma in a large european cohort. *Hepatobiliary and Pancreatic Diseases International*.

[81] Shinozaki A., Sakatani T., Ushiku T. (2010). Downregulation of MicroRNA-200 in EBV-associated gastric carcinoma. *Cancer Research*.

[82] Zhou M., Liu Z., Zhao Y. (2010). MicroRNA-125b confers the resistance of breast cancer cells to paclitaxel through suppression of pro-apoptotic Bcl-2 antagonist killer 1 (Bak1) expression. *Journal of Biological Chemistry*.

[83] Lin C.-C., Jiang W., Mitra R., Cheng F., Yu H., Zhao Z. (2015). Regulation rewiring analysis reveals mutual regulation between STAT1 and miR-155-5p in tumor immunosurveillance in seven major cancers. *Scientific Reports*.

[84] Hwang J. H., Voortman J., Giovannetti E. (2010). Identification of microRNA-21 as a biomarker for chemoresistance and clinical outcome following adjuvant therapy in resectable pancreatic cancer. *PLoS ONE*.

